# Clonal Hypereosinophilic Syndrome: Two Cases Report in Black Men from Sub-Saharan Africa and Literature Reviews

**DOI:** 10.5402/2011/974609

**Published:** 2011-03-22

**Authors:** Kodjovi Messie, Ahoefa Vovor, Irenee Messanh Kueviakoe, Levi kankoe Sallah, Kossi Agbetiafa, Akuete Yvon Segbena

**Affiliations:** ^1^Medical Faculty and Hematology Laboratory of the Campus Teaching Hospital of Lomé, Lomé, Togo; ^2^Medical Faculty and Hematology Laboratory of Tokoin Teaching Hospital of Lomé, Lomé, Togo

## Abstract

The first case is about a man of 60 years old suffering of hypereosinophilic syndrome (HES) developed since 1998. He presented chronic cough, insomnia, and negative parasitical test. We observed hypereosinophilia and fibroblastic hyperplasia at the bone marrow biopsy. Initially, hydroxyurea and **α**-interferon treatment failed. We proposed to him imatinib mesylate in May 2003. The FIP1L1-PDGFRA gene was detected. The second case is about a man of 34 years old seen in March 2002. First investigation concluded to CML. Progressively, eosinophil cells increased, and complications occurred as oedema syndrome, dyspnoea, and parietal chronic endocarditic fibrosis associated with pericarditis. In addition, a bowel obstruction happened and was cured by surgery. Bcr-abl fusion was negative, and FIP1L1-PDGFRA gene was detected after and imatinib mesylate was given. Actually, endocarditic fibrosis decreased. The two patients are in haematological and cytogenetic remission. We concluded that clonal HES is present in Africa, and imatinib mesylate is effective.

## 1. Introduction

Hypereosinophilia (HE) is defined as a number of eosinophil granulocytes equal or greater than 0.5  ×  10^9^/liter or 500/mm^3^ of circulating blood [1]. It may result from numerous causes most of which can be identified by throughout patient's history. In the majority of the cases, the HE is secondary to an infection (mostly by a parasite), an environmental allergen, or a medication. In few cases, the HE is caused by a systemic disease such as a vasculitis. In some situations no etiology is found to the HE even after an exhaustive investigation. This type of eosiphilia has been called hypereosinophilic syndrome (HES) or Chusid's syndrome since 1975 [2]. The criteria of WHO classification of chronic myeloproliferative diseases (2002) for hypereosinophilic syndrome (HES) are the following: eosinophilia >1.5  ·  10^9^/L for >6 months and by end-organ damage, commonly endomyocardial fibrosis, skin lesions (angioedema, urticaria), thromboembolic disease, pulmonary, lesions and central nervous system dysfunction. Associated with polyclonal increased in immunoglobulins including IgE, splenomegaly 40%, anemia 50%, and neutrophila with left shift frequent, platelets may be normal, decreased, or increased. Bone marrow hypercellular with 25–75% eosinophils with left shift [3].

Because of its poor prognosis [4], the challenge is to make an early diagnosis. Even though Bain's criteria are still used to characterize the HES, recent progress in molecular biology has brought a new light into the pathophysiology and the treatment of this syndrome. The discovery of the FIP1L1-PDGFRA [5] gene was an important step toward the understanding of the disease. Different subtypes of the HES have been reported: the myeloproliferative type that responds well to imatinib mesylate, the lymphoproliferative subtype, and other syndromes with different clinically and with separate therapeutic orientations. Imatinib, an tyrosine kinase inhibitor has successfully been used [6] to treat the FIP1L1-PDGFRA positive hypereosinophilic syndromes (HES). This subtype of HES has not been well documented amongst the sub-Saharan African populations [7].

We are reporting in this study 2 cases of FIP1L1-PDGFRA HES also known as clonal hypereosinophilic syndrome, diagnosed in the clinical hematology unit of the Laboratory services of the Campus teaching hospital of Lomé in Togo. The objectives of this study are to describe the clinical features of these 2 cases, to underline the diagnostic and therapeutic difficulties giving our context, and to propose a strategy to improve the diagnosis and treatment of patients with clonal HES.

## 2. Case Report

**Case 1**

Mr. A. Y. a 57-year-old Togolese man was referred to the hematology unit in June 1999 for a chronic dry cough and a hypereosinophilia. His past medical and family history is negative for atopic diseases, diabetes, and hypertension. He carries the AC hemoglobin type and mentioned a hepatitis A infection in 1963 and two surgeries for a disc hernia.

Since December 1998, he has been having a dry cough, refractory to cough suppressants. He was seen in cardiology in May 1999, where a clinical wokup was significant for an hypereosinophilia of 4680/mm^3^. His chest X-Ray and chemistry panel including BUN, creatinine, and blood glucose were negative. He was treated with antihistamine and expectorant agents without any success. He was referred to hematology in June 1999. He was well appearing with no acute distress. His vital signs and physical exam were within normal limits, mainly there were no hepatosplenogaly or lymphadenopathy. In addition to the tests performed in cardiology, a marrow aspiration showed a granular hyperplasia (82%) with a central hypereosinophilia of 37%. Investigations to detect a possible parasite infection or an atopic cause were negative.

On his request, Mr. A. Y. was transferred in November 1999 in Paris for further testing. The search for parasites done in Lome was completed in Paris by series of serology which was negative for trichinosis, toxocariasis, anguillilosis, distomatosis, and filariasis. Tumor markers: ACE, CA-19.9, PSA, and alpha fetoprotein were within the normal ranges and the chest X-ray, EKG and echocardiogram, lung function tests, and CT of the chest and the abdomen were normal.

The diagnosis of a myeloproliferative syndrome type hypereosinophilia was adopted and Mr. A. Y. was started on hydroxyurea one tablet of 500 mg a day and allopurinol 300 mg a day.

On 8th May 2000, the patient was seen back in Lome with no change in the eosinophil count. It was agreed to increase the hydroxyurea to 2 tablets of 500 mg a day. This was of no effect on the eosinophil count but help to normalize transiently the patient's platelet count. Clinically, the patient stated that the cough did not improve; in fact, it was getting worse and was disturbing his sleep. On July 11th 2000, the hydroxyurea was changed to alter 2 or 3 tablets on days. In December 2000, the patient suffered from an oral mycosis and was treated with mouthwashes with a mixture of chlorexidine, amphotericin B and sodium bicarbonate solution. He also complained of a pruritis, and in January 2001, the patient had an onychomycosis due to trichophyton rubrum and which was treated with terbinafin, hexomedine, and colchicine. Following the dermatologists advice, the hydroxyurea was temporary discontinued. The CBC of February and April 2001 showed an eosinophil count of 7220 and 7880/mm^3^, respectively. The treatment with hydroxyurea was not resumed, because it was not efficient before the therapeutic window. The patient general condition did not improve; he was, in fact, loosing weigh and also because the subsequent CBCs showed an anemia on top of the hypereosinophilia. In February 2002, the patient present with an elbow mass of the size of a gulf ball, firm, associated with a right groin lymphadenopathy which was biopsied and the biopsy report concluded to a nonspecific adenitis.

The molecular study was negative for a T lymphocytes clone; the marrow biopsy revealed an important fibroblastic hyperplasia and a diffuse increase of reticular network. A treatment with Alpha interferon at 3 IU three times a week was started on August 02, 2002. The CBC and the liver function tests were checked every week. After 2 weeks of treatment, Mr. A. Y. noticed an improvement of the cough but complained of tiredness. This treatment was continued until October despite episodes of bodyache and insomnia.

A paper in Lancet has reported the efficacy of Imatinib mesylate in 5 patients with HES [8]. After discussing, we proposed to our patient to try this medication. After one month on imatinib at 100 mg twice daily, eosinophilia passed from 3956/mm^3^ to 78/mm^3^. The cough and the pruritis improved, but because of the low WBC, an antibiotic coverage was prescribed, and the imatinib discontinued. After 5 months, the patient reported a steady improvement of his symptoms and a better sleep quality.

An article in the NEJM in Marsh 2003 by Cools et al. [9] reported that a tyrosine kinase which results from the fusion of the PDGRA and FIP1L1 genes was the target of imitanib in patients with HSE. In blood sample molecular analysis of our patient, the PDGRA-FIP1L1 was found positive; this fitted our patient in the myeloproliferative PDGRA-FIP1L1 HES group as described by Cools et al. [9]. Six subsequent evaluations for the fusion gene were negative, showing a complete cytogenetic remission. The last evaluation for the fusion gene 17 months was also negative. Actually, imatinib was reduced to 1 tablet a day.

In conclusion, the diagnosis of PDGRA-FIP1L1 positive hypereosinophilic syndrome myeloproliferative subtype was made for this patient.

**Case 2**

Mr. M. K. a 32-year-old was seen in June 2002 for an eosinophil count of 18 348/mm^3^ on his annual checkup. His past medical history is negative, and he denied any family history for atopy, has smoked a half pack of cigarettes a day for 10 years, and has stopped smoking since 2001. His vital signs were within normal limit, and the clinical examination was normal except a diminished breath sound on the left lung, there was no hepatosplenomegaly. The stools and urine study for parasites as well as the serologic tests for the common parasites were negative. A white blood count a week before this visit demonstrated a leukocytosis of 205,400/mm^3^. A marrow aspiration showed a granulocytic hyperplasia with an important eosinophil content (19%). All these findings were consistent with a chronic myelocytic leukemia (CML) in the chronic phase. The patient was started on hydroxyurea six tablets of 500 mg and allopurinol 300 mg a day. Soon after staring these medications, the patient presented with a pitting edema of the lower extremities, unsteady gait, a right hypoacousia with hypoesthesia of the right side of his face. The neurology and the ENT evaluations did not find any abnormalities. The initial treatment failed to bring up a steady hematological remission, and a second marrow aspiration on 01/03/2003 was still show features compatible with the diagnosis of CML. We replaced hydroxyurea by cytosine arabinoside known to be a more potent drug, 50 mg intramuscular injection. Since 01/15/2003, the patient begun to develop complications imputable to the hypereosiphilic syndrome.

- An ascitis and hepatomegaly [10, 11] (on clinical exam and also shown by the abdominal echography of 01-27-2003), associated with edema and decreased prothrombin time (PT) 38% (range 70%–100%). These symptoms were ruled to be due to liver cirrhosis; therefore, the cytosine arabinoside was stopped because of its potential of liver toxicity and toxic encephalopathy. But because the white cell count was increasing rapidly, hydroxyurea was resumed at smaller doses. Meanwhile, a treatment with vitamin K resulted in bringing the PT up to 70%.
- A recurrent anemia [12] which warranted many transfusions of iso-group and iso-Rhesus blood units.
- A permanent shortness of breath and generalized edema in March 2003. A cardiology workup discovered a cardiomyopathy [13] with a small pericaditis [14] and a significantly impaired left ventricular compliance. The previous liver abnormalities were probably the result of a right heart failure. A treatment with diuretics and vasodilators (furosemide, spironolactone/altizide, and isosorbide dinitrate) was added to hydroxyurea.

In addition to the above complications, the gait unsteadiness and the right hypoacousia persisted.

Other investigations including a CT of the brain with contrast, HIV and HBV tests, and kidney tests were normal. The patient had a bowel obstruction and was hospitalized for surgical treatment for 4 months. Little before the surgery, thorough investigations demonstrated that there was no Philadelphia chromosome and made the diagnosis an HES positive for the FIP1L1-PDGFRA fusion gene [9]. The patient was started on imatinib on 11/06/2003, 2 tablets of 100 mg a day. The CBC before treatment showed eosinophils 7891/mm^3^. After 3 weeks of treatment, a remission was achieved as shown by the eosinophil count of 280/mm^3^. Imatinib was then reduced to 100 mg a day for the next 16 months and 6 days out of 7 for the following 12 months. These dosages were sufficient to maintain the good and stable cytological response.

Actually, the patient is in a general good health. The CBC revealed eosinophils at 128/mm^3^. The abdominal echography demonstrated that the hepatomegaly and the distension of the sus-hepatic veins from the cardiac liver were stable. The cardiac Doppler echography noted an hemodynamic improvement despite the persistence of the cardiomyopathy. The chest X-ray was normal. The FIP1L1-PDGFRA fusion gene was still present in 3 subsequent molecular biology testing performed in 2 years. The dosage of imitanib was, therefore, increased to 200 mg a day. The last control showed that the FIP1L1-PDGFRA fusion gene was negative. A possible resistance genes was not found.

In summary, an HES of the myeloproliferative type [15] with FIP1L1-PDGFRA fusion gene was the final diagnosis for this patient.

## 3. Discussion

The analysis of these cases of hypereosinophilic syndrome in 2 Black Africans brings up several points related to the following:

- the definition and the disease terminology, which are not definite and are evolving with the new scientific developments,
- the epidemiology, these 2 cases are reports of a disease rarely studied [7] or described in sub-Saharan Africa,
- the diagnostic workup that requires tools not always available in that part of the world,
- lastly, to the treatment which has improved from recent innovations.

### 3.1. Definitions and Nomenclatural Concepts

Our first patient was initially seen in 1999. Essential hypereosinophilia was exclusively defined by Chusid's criteria [2] formulated since 1975. Based on these criteria a diagnosis of EHS would have been satisfactory for that patient. In today's practice, because of the recent progress, the definition of HES became disputable. Since the discovery of the FIP1L1-PDGFRA fusion gene [9] in 2003, is it acceptable to call this disease “essential” while that gene is strongly implicated in the pathophysiology of that type of hypereosinophilia? Many authors have responded by the negative to that question and have recommended to reserve the use of the qualifiers *idiopathic* and *essential* for the cases for which no molecular abnormalities [16] or other etiology were identified [17]. Our patients had the FIP1L1-PDGFRA fusion gene, and, therefore, their diagnosis was clonal hypereosinophilic syndrome with molecular rearrangement PDGFR*α*. This exemplifies the difficulty to study heterogeneous groups of idiopathic diseases for their pathophysiologic mechanisms could be later identified. The most recent World Health Organization classification of the eosinophilic syndromes is from 2002 [3]. This illustrates the delay between scientific progress and their use in classifying diseases. Furthermore, the Chusid's criteria suggested that patients should have an organ involvement, but Mr. A. Y. did not present any, and we did not find any objective pulmonary cause for his chronic cough. His case is close to recent studies that found the FIP1L1-PDGFRA fusion gene in patients who did not have any systemic manifestations. It appears important that the third criterion of Chusid's syndrome be readjusted.

### 3.2. Epidemiologic Considerations

HES is a rare pathology and cases with positive FIP1L1-PDGFRA are even more uncommon based on the data available, the fusion gene is found only in 17% to 56% of the time. Both of our patients had the clonal HES and were the only 2 cases diagnosed in 10 years (1998–2007) in the Laboratories service of the Campus University Hospital. We did not any other description of the disease in African medical literature [7]. One reason for this is the lack of advanced investigative tools such as molecular biology techniques to identify the FIP1L1-PDGFRA fusion gene. Our study confirms the putative notion that the clonal HES does not have a predilection for a race. A greater number of cases are needed before the rates of race repartition can be eventually established. Our results are also in concordance with the literature with regards to the sex ratio, and clonal HES has a clear male predominance [18, 19]; from all the cases reported, there is a male to female sex-ratio of 22 : 1. This may be the result of a genetic differences or hormonal influences on the break point on the chromosome 4 and the expression of the fusion product. This type of inegality has been also documented for PDGFR*β* [20]. For the few female cases of FIP1L1-PDGFRA, the systemic involvement was minimal if not absent [9]. This may have play a role in the low prevalence of the disease in women, because they were pauci- or asymptomatic. The disease has been reported in broad range of age 16–72 years old. Our 2 patients were 32 and 56 years old, respectively. It seems that clonal HES is not a disease of young adult only as suggested by some previous data [2].

M. K., before been treated with imitanib, had a poor prognosis because of the hypertrophic cardiomyopathy [21] with congestive heart failure. His condition was greatly improved with the treatment despite the fact that the myocardial fibrosis did not completely resolve. For the FIP1L1-PDGFRA positive cases, the earlier the diagnosis is made and the treatment started, the lower the mortality and morbidity [22]. One element seems prejudicial in the case of M. K., which is the persistence of the FIP1L1-PDGFRA at subsequent molecular evaluations despite the imitanib therapy. This is reported as a sign of poor prognosis [9, 22, 23], because there is a risk for transformation into an eosinophilic leukemia which is a highly fatal disease. We are going to investigate the possibility of the 2149 C→T mutation of the PDGFRA sequence which was found in 3 patients with resistant to imitanib [9, 22].

### 3.3. The Diagnosis Approach

For our first patient, the possibility of an HES was considered after a thorough investigation including a test for the Philadelphia chromosome [24] did not find any causes for the eosinophilia. When the FIP1L1-PDGFRA fusion gene was later described, we were able to test our patient for it. 

For the second patient (seen initially in June 2002), we considered the possibility of a chronic myelocytic leukemia (CML), which we could not rule out until the test for the Philadelphia chromosome was performed in August 2003 by a french laboratory. The negative result made the possibility of a clonal HES likely. The delay could have been avoided if we had all the diagnostic tools available locally. M. K. presented some neurological manifestations at the end of 2002. He had an unsteadiness of the gait more while walking. A neurology consult did not find any pathology; therefore, we considered this symptom to be related to the hypereosinophilia [25, 26]; such nervous system involvement has been reported in 23% of the cases. M. K. also had an eosinophilic cardiopathy [27] that was described in 29% of the patients.

Both of our cases presented with a very high eosinophil count (more than 7000/mm^3^) during the course of the disease and were found positive for the FIP1L1-PDGFRA gene. A study [28] of a cohort of 35 eosinophilia suggested that patients with 3000 or more eosinophils/mm^3^ were more likely to be positive for the FIP1L1-PDGFRA gene. The same study showed that 100% of FIP1L1-PDGFRA positive patients had a high level of serum tryptase, and that 85% of patients with HES and an elevated serum tryptase were FIP1L1-PDGFRA fusion gene positive. An elevated serum tryptase seems a good sensitive and specific marker for the presence of the FIP1L1-PDGFRA rearrangement and, therefore, for clonal HES. We did not study the serum tryptase in our patients. It has also been reported that elevated serum vitamin B12, LDH, and leukocyte alkaline phosphatase (LAP) are good criteria for myeloproliferative type HES. This was not proven in our patients moreover A. Y. had a normal LDH level in June 1999 while the disease was evolutive.

### 3.4. Treatment

Our patients, like 66% of the cases in the literature, were treated with other drugs before imatinib. With a dosage of 200 mg a day of imatinib, both patients responded well, and a significant decrease in their eosinophilia was achieved: 7891 to 280/mm^3^ in 22 days for M. K. and 3956 to 78/mm^3^ in 37 days for A. Y. A dosage of 100 mg a day is now recommended for the treatment of FIP1L1-PDGFRA positive eosinophilia. Some authors proposed a trial with that drug even when the fusion gene is not present, based on reports by Walz et al. [29] and Salem et al. [30] of patients with FIP1L1-PDGFRA negative HES that responded well to imatinib. This suggests that the possibility of other cytogenetic abnormalities which are not yet identified.

In both of our patients, the treatment with imatinib resulted in also a significant clinical improvement. M. K. showed a remarkable amelioration of his cardiac function; his cardiomyopathy which was well advanced before the diagnosis of the clonal eosinophilia would have needed more aggressive treatments even cardiac surgery back in the days when imatinib was not used. In A. Y., the chronic cough and the pruritus have totally subsided. Despite this globally good outcomes, the persistence of the FIP1L1-PDGFRA fusion gene, the elevated LDH, the high serum uric acid in M. K. may be indicative of a persisting cellular proliferation. That patient is being closely monitored because of concerns of a transformation into eosinophilic leukemia.

The main issue with the treatment in our context of practice is its cost and availability. 60 tablets of 100 mg costs 1287 Euros (800,000 francs CFA, more than 2 times the minimum annual salary in Togo) and the medication has to be ordered from abroad. This explain the delay in the starting of the treatment for both of our patients and the interruption of treatment in February 2007 for A. Y.

## 4. Conclusion

These 2 cases of clonal hypereosinophilic syndrome underlined the difficulties of the diagnostic workup of the many differentials of hypereosinophilia in sub-Saharan Africa [7]. Essential hypereosinophilic syndrome, an already rare condition, is certainly under-diagnosed in Africa. This study also allowed us to review the progress made in the diagnosis and treatment of the disease. Our 2 patients benefited from the recent identification of the FIP1L1-PDGFRA fusion gene [9] as major role player in the pathophysiology of clonal HES, by activation of a tyrosine kinase receptor. Imatinib mesylate a tyrosine kinase inhitor which has been used since 2001 for clonal HES was proven efficient in both of our patients. 

**Figure 1:**
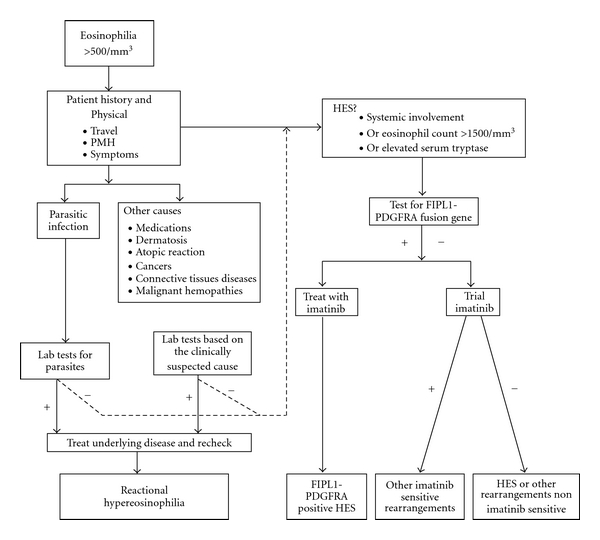
Clinical workup of hypereosinophilia + positive results or outcome, − negative results or outcome, HES hypereosinophilic syndrome PMH past medical history.

After analyzing our 2 cases and reviewing the medical literature available, we are suggesting the diagnostic strategy to guide the workup of hypereosinophilic syndromes as presented in Figure 1.
